# Notch, IL-1 and Leptin Crosstalk Outcome (NILCO) Is Critical for Leptin-Induced Proliferation, Migration and VEGF/VEGFR-2 Expression in Breast Cancer

**DOI:** 10.1371/journal.pone.0021467

**Published:** 2011-06-23

**Authors:** Shanchun Guo, Ruben R. Gonzalez-Perez

**Affiliations:** Department of Microbiology, Biochemistry and Immunology, Morehouse School of Medicine, Atlanta, Georgia, United States of America; University of Pennsylvania, United States of America

## Abstract

High levels of pro-angiogenic factors, leptin, IL-1, Notch and VEGF (ligands and receptors), are found in breast cancer, which is commonly correlated with metastasis and lower survival of patients. We have previously reported that leptin induces the growth of breast cancer and the expression of VEGF/VEGFR-2 and IL-1 system. We hypothesized that Notch, IL-1 and leptin crosstalk outcome (NILCO) plays an essential role in the regulation of leptin-mediated induction of proliferation/migration and expression of pro-angiogenic molecules in breast cancer. To test this hypothesis, leptin's effects on the expression and activation of Notch signaling pathway and VEGF/VEGFR-2/IL-1 were determined in mouse (4T1, EMT6 and MMT) breast cancer cells. Remarkably, leptin up-regulated Notch1-4/JAG1/Dll-4, Notch target genes: Hey2 and survivin, together with IL-1 and VEGF/VEGFR-2. RNA knockdown and pharmacological inhibitors of leptin signaling significantly abrogated activity of reporter gene-luciferase CSL (RBP-Jk) promoter, showing that it was linked to leptin-activated JAK2/STAT3, MAPK, PI-3K/mTOR, p38 and JNK signaling pathways. Interestingly, leptin upregulatory effects on cell proliferation/migration and pro-angiogenic factors Notch, IL-1 and VEGF/VEGFR-2 were abrogated by a γ-secretase inhibitor, DAPT, as well as siRNA against CSL. In addition, blockade of IL-1R tI inhibited leptin-induced Notch, Hey2 and survivin as well as VEGF/VEGFR-2 expression. These data suggest leptin is an inducer of Notch (expression/activation) and IL-1 signaling modulates leptin effects on Notch and VEGF/VEGFR-2. We show for the first time that a novel unveiled crosstalk between Notch, IL-1 and leptin (NILCO) occurs in breast cancer. Leptin induction of proliferation/migration and upregulation of VEGF/VEGFR-2 in breast cancer cells were related to an intact Notch signaling axis. NILCO could represent the integration of developmental, pro-inflammatory and pro-angiogenic signals critical for leptin-induced cell proliferation/migration and regulation of VEGF/VEGFR-2 in breast cancer. Targeting NILCO might help to design new pharmacological strategies aimed at controlling breast cancer growth and angiogenesis.

## Introduction

Notch, IL-1 and leptin are notorious pro-angiogenic factors whose over-expression characterized growth of breast cancer, metastasis and poor prognosis. Notch signaling is essential for angiogenesis, functions in an enormous diversity of developmental processes, and its dysfunction is implicated in many cancer types. Notably, Notch and leptin are pro-proliferation factors for many cell types [1], [2], [3], [4]. Expression of Notch1 and its ligand, JAG1, is associated with the poorest breast cancer survival and increased levels of VEGFR-2 [5], [6]. Notch signaling is activated by ligand binding expressed by adjacent cells, followed by ADAM/TACE and γ-secretase cleavages that produce the Notch intracellular domain (NICD). NICD interacts with nuclear RBP-Jk or CBF1/Su(H)/Lag-1 (CSL) family of transcription factors to activate Notch target genes (*i.e.* survivin, Hey2) [3]. The GSIs or small molecule inhibitors of γ-secretase abrogate Notch actions in cancer, but they show many side-effects [3].

Increased levels of IL-1 system components (ligands: IL-1α/-β; antagonist: IL-1Ra and receptor: IL-1R tI), are found in breast cancer [7]. IL-1 binding to IL-1R tI activates p38 MAPK and NF-κB signaling pathways to induce VEGF [8] and VEGFR-2 expression (Gonzalez-Perez; Manuscript in preparation). IL-1 signaling promotes breast cancer development. Indeed, blockade of IL-1R tI with IL-1Ra has shown to negatively impact on angiogenesis and growth of human breast cancer xenografts in mouse models [7], [9].

Obesity, a pandemic in the US, is associated with more than 100,000 incidents of cancer in the US every year particularly cancers of the breast, colon, and endometrium. Obese breast cancer patients have increased mortality compared to non-obese patients [10]. Leptin is mainly produced by adipose tissue, but also abnormally expressed together with its receptor (OB-R) in BC cells [11], [12]. Higher levels of leptin are found in female, postmenopausal women and obese individuals. Accumulating evidence show leptin is an important pro-angiogenic, pro-inflammatory and mitogenic factor [13], [14], whose actions are reinforced through crosstalk with cytokines/growth factors [3], [5], [15], [16], [17], [18]. Leptin signals through activation of its receptor, OB-R that induces several canonical (JAK2/STAT; MAPK/ERK1/2 and PI-3K/AKT1) and non-canonical signaling pathways (PKC, JNK and p38 MAP kinase) to exert its biological effects in food intake, energy balance, and adiposity as well as in the immune and endocrine systems [19], [20].

We have previously shown that leptin signaling could give an additional advantage to breast cancer by up-regulating VEGF/VEGFR-2 before hypoxia is manifested [18]. Remarkably, VEGF/VEGFR-2 directly regulates tumor angiogenesis and also works as an essential autocrine/paracrine process for breast cancer cell proliferation and survival [5]. Moreover, it was recently reported that leptin is able to phosphorylate VEGFR-2 in absence of VEGF in human endothelial cells [21]. Increasing evidence suggest leptin signaling could be an important link between breast cancer incidence/growth and obesity. Restriction of caloric intake (25%) reduces 7,12-dimethylbenz[a]anthracene (DMBA)-mammary tumor incidence in rats [22]. Similar effects were found using a pegylated leptin peptide receptor antagonist (PEG-LPrA2) for inhibition of leptin signaling in lean and diet-obesity-induced mice treated with DMBA [Gonzalez-Perez, Manuscript in preparation]. Furthermore, the elegant work of Dr. Cleary's group has shown that obese mice with deficiency in leptin signaling (*ob/ob* or *db/db*) show a significantly lower incidence of mammary tumors than their lean littermates. On the other hand, MMTV-TGF-α mice have a proclivity to develop mammary tumors, but when crossed with leptin/OB-R deficient mice, there is a reduced incidence of tumors in their progeny [23], [24]. Furthermore, we previously demonstrated that the disruption of leptin signaling with PEG-LPrA2 markedly reduced the growth of tumors in mouse models of syngeneic and human breast cancer xenografts [25], [26]. PEG-LPrA2′s effects were accompanied by a significant decrease in VEGF/VEGFR-2, IL-1R tI, cyclin D1 and PCNA levels [25], [26].

In line with these data, we recently provided a comprehensive mechanism for leptin-mediated regulation of VEGF in breast cancer cells [27]. In addition, we have shown that leptin increases protein and mRNA levels of all components of the IL-1 system in breast cancer [18]. Leptin upregulation of VEGF promoter was linked to AP1, HIF-1 and NF-κB [27] meanwhile leptin-mediated activation IL-1α promoter was linked to SP1 and NF-κB transcription factors [18]. Interestingly, leptin upregulation of VEGF/VEGFR-2 in breast cancer was partially mediated by IL-1/IL-1R tI signaling [18]. However, the molecular mechanisms of leptin pro-angiogenic actions in breast cancer are still not fully understood. Since Notch signaling and its crosstalks with many signaling pathways play an important role in breast cancer cell growth, migration, invasion, metastasis as well as angiogenesis [3], we hypothesized that a crosstalk between Notch, IL-1 and leptin signaling could impact on the expression of pro-angiogenic molecules, and induction of cell proliferation and migration in breast cancer. To test this hypothesis, we used mouse mammary cancer cell lines (4T1, EMT6 and MMT) that differentially respond to leptin and express OB-R, VEGF/VEGFR-2 and IL-1/IL-1R tI [27]. Our results show for the first time that leptin is a regulator of Notch expression and activation in breast cancer. Leptin-induction of cell proliferation/migration along with VEGF/VEGFR-2 was highly dependent of a novel unveiled crosstalk between Notch, IL-1 and leptin (NILCO) in breast cancer cells.

## Results

### Leptin induces the expression of Notch receptors and ligands in breast cancer cells

**Figure 1:**
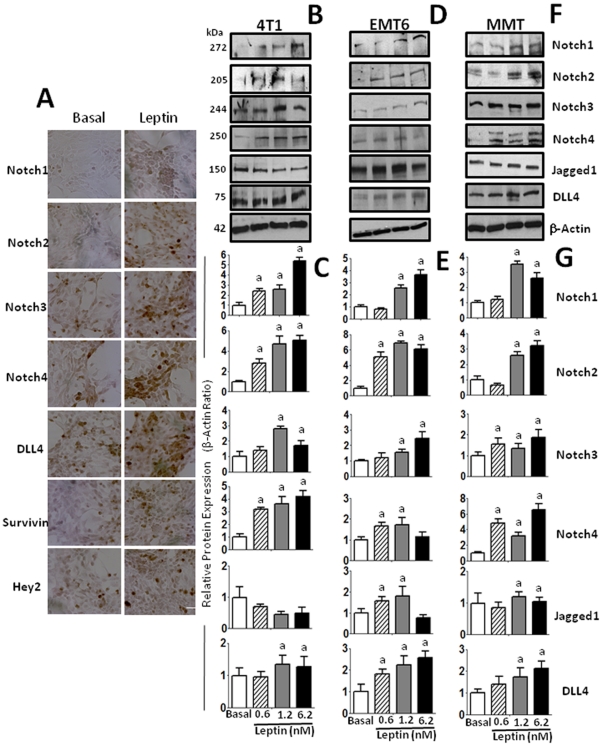
Leptin induces the expression of Notch and its molecular targets in mammary cancer cells. Representative results of leptin-dose effects on increase in protein levels of Notch (ligands and receptors) and molecular targets as determined by immunocytochemistry in 4T1 cells (A) and western blot (WB) in 4T1 (B and C), EMT6 (D and E) and MMT (F and G) cells. Cells were cultured for 24 h and leptin dose-induced (0, 0.6, 1.2 and 6.2 nM) effects were determined as described (See M &M). The WB results were normalized to β-actin as loading control and densitometric analysis of bands was carried-out with the imageJ software. (a) *P<0.05* when comparing levels of protein to control (basal). Data (mean ± standard error) representative results derived from a minimum of 3 independent experiments. Bar = 200 µm.

Leptin upregulation of Notch component expression was initially determined by immunohistochemistry. Figure 1A shows representative pictures for leptin-mediated increase in protein levels of Notch receptors (Notch1,2,3,4), ligand (DLL-4) and targets (survivin, Hey2) in 4T1 cells. Western blot analyses further assessed these findings in 4T1 cells. Leptin generally induced protein expression of Notch receptors (Notch1∼4) and ligands, (JAG1, DLL-4) in a dose-response manner in the mouse mammary cancer cells investigated (Figure 1 B–G). However, the levels of JAG1 expression were downregulated by leptin in 4T1 cells, but DLL-4 was upregulated by leptin in all cells. Leptin differentially up-regulated Notch receptors in mammary cancer cells. In 4T1 cells leptin-mediated induction of Notch shows the following pattern: Notch1, Notch2> Notch4>Notch3 and DLL-4 (Figure 1B–C). In contrast, leptin induction of Notch in EMT6 cells was: Notch2>Notch1> Notch3>Notch4 and DLL-4>JAG1 (Figure 1D–E). On the other hand, the pattern of leptin-induction of Notch in MMT cells was: Notch4>Notch1, Notch2>Notch3 and DLL-4>JAG1 (Figure 1F–G). The biological relevance of the leptin-relative induction levels of Notch receptors in each cell line remains to be investigated. We also observed similar leptin effects on Notch signaling when we used two human breast cancer cell lines, MCF-7 and MDA-MB-231 (data not shown).

### Leptin-induced Notch signaling up-regulates Notch mRNA and target genes: survivin and Hey2

**Figure 2:**
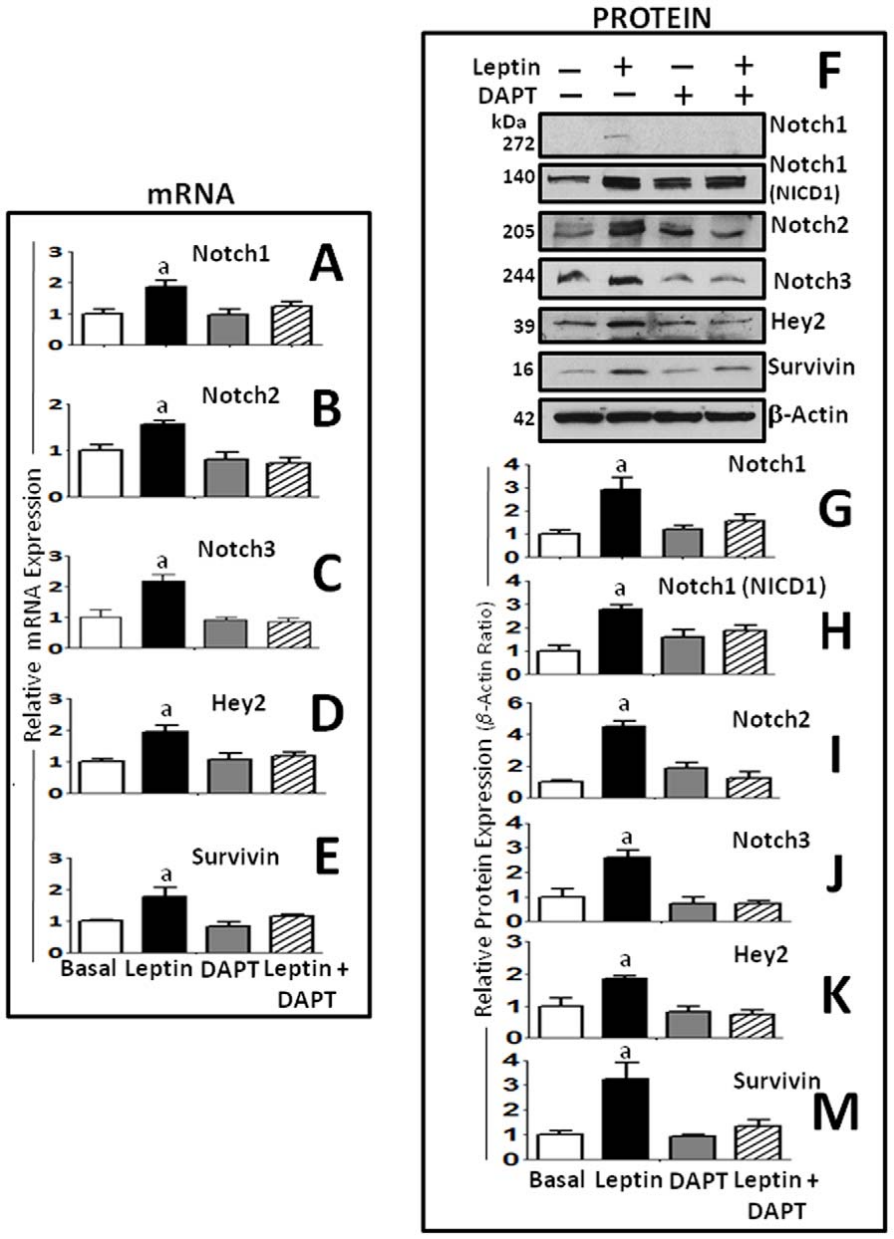
Leptin-mediated upregulation of protein and mRNA levels of Notch receptors and targets in mammary cancer cells are abrogated by a γ-secretase inhibitor (DAPT). Leptin-induced transcriptional expression of Notch receptors and targeted genes was abrogated by DAPT in 4T1 cells. Levels of Notch receptors mRNA (A, Notch1; B, Notch2 and C, Notch3) and targeted genes (D, Hey2 and E, survivin) as determined by real-time RT-PCR. GAPDH was used as internal control. (F–M) Representative results of leptin upregulation of protein levels of Notch1–3 and activated NICD1 as well as Notch targeted genes, Hey2 and survivin in 4T1 cells as determined by western blot (WB). The WB results were normalized to β-actin as loading control and densitometric analysis of bands was carried-out with the imageJ software for (G) NICD1; (H) Notch1; (I) Notch 2; (J) Notch 3; (K) Hey2 and (M) survivin. 4T1 cells were cultured in a medium containing 0 or 1.2 nM leptin for 24 h. (a) *P<0.05* when comparing levels of mRNA or protein to control (basal). Data (mean ± standard error) representative results derived from a minimum of 3 independent experiments.

To further investigate the effects of leptin on the transcriptional expression of Notch, the next series of experiments were made with 4T1 cells. In these cells leptin up-regulated the transcriptional expression of Notch (Notch1-3, DLL-4) and target genes (Hey2, survivin) (Figure 2A). Interestingly, leptin-induced effects on transcriptional/translational regulation of the above molecules were abrogated by DAPT (an inhibitor of Notch activation) (see Figure 2A–B).

### Leptin induced expression of Notch and its targets is abrogated by CSL siRNA

**Figure 3:**
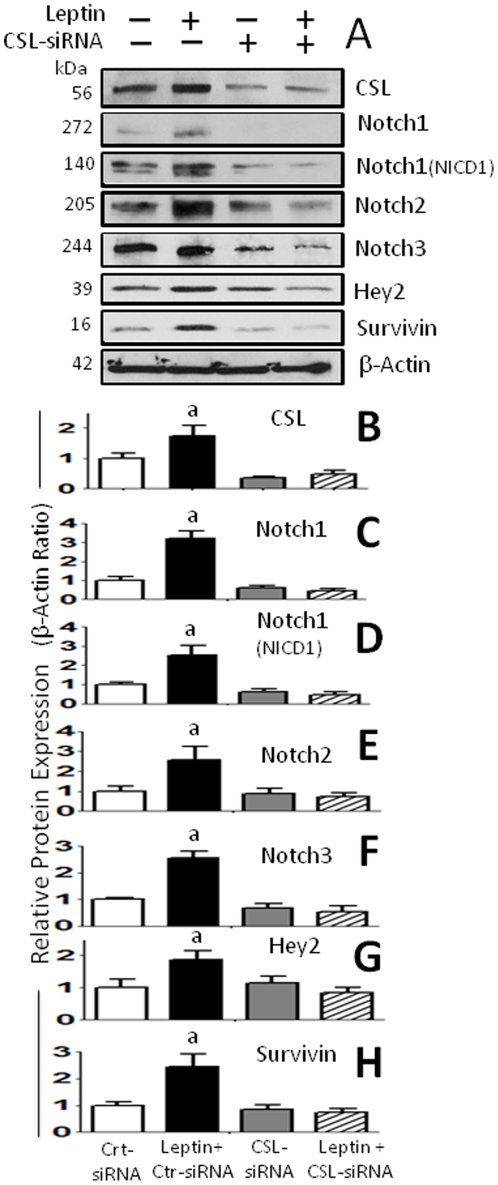
Leptin-induced expression of Notch receptors and targeted genes in mammary cancer cells are abrogated by CSL-RNA knockdown. (A) Representative results of CSL-siRNA inhibition on leptin upregulation of protein levels of Notch 1-3, activated NICD1 and Notch targeted genes, CSL, survivin and Hey2 in 4T1 cells as determined by western blot (WB). The WB results were normalized to β-actin as loading control and densitometric analysis of bands was carried-out with the imageJ software for (B) CSL; (C) Notch1; (D) NICD1; (E) Notch 2; (F) Notch 3; (G) Hey2 and (H) survivin. Cells cotransfected with siRNA oligonucleotides (CSL-siRNA or control-siRNA) were treated with leptin (0 and 1.2 nM) for 24 h. (a) *P<0.05* when comparing protein levels to cells treated with control-siRNA (basal) or CSL-siRNA. Data (mean ± standard error) representative results derived from a minimum of 3 independent experiments.

In order to investigate whether leptin-induced regulation of Notch/targets is also dependant of CSL regulation CLS-siRNA was used. The addition of CSL-siRNA also significantly inhibits leptin-induced expression of Notch receptors and targets, Hey2 and survivin (Figure 3). Interestingly, leptin also up-regulated CSL protein expression (Figure 3A,B).

### Several leptin-induced signals are involved in CSL transcription

**Figure 4:**
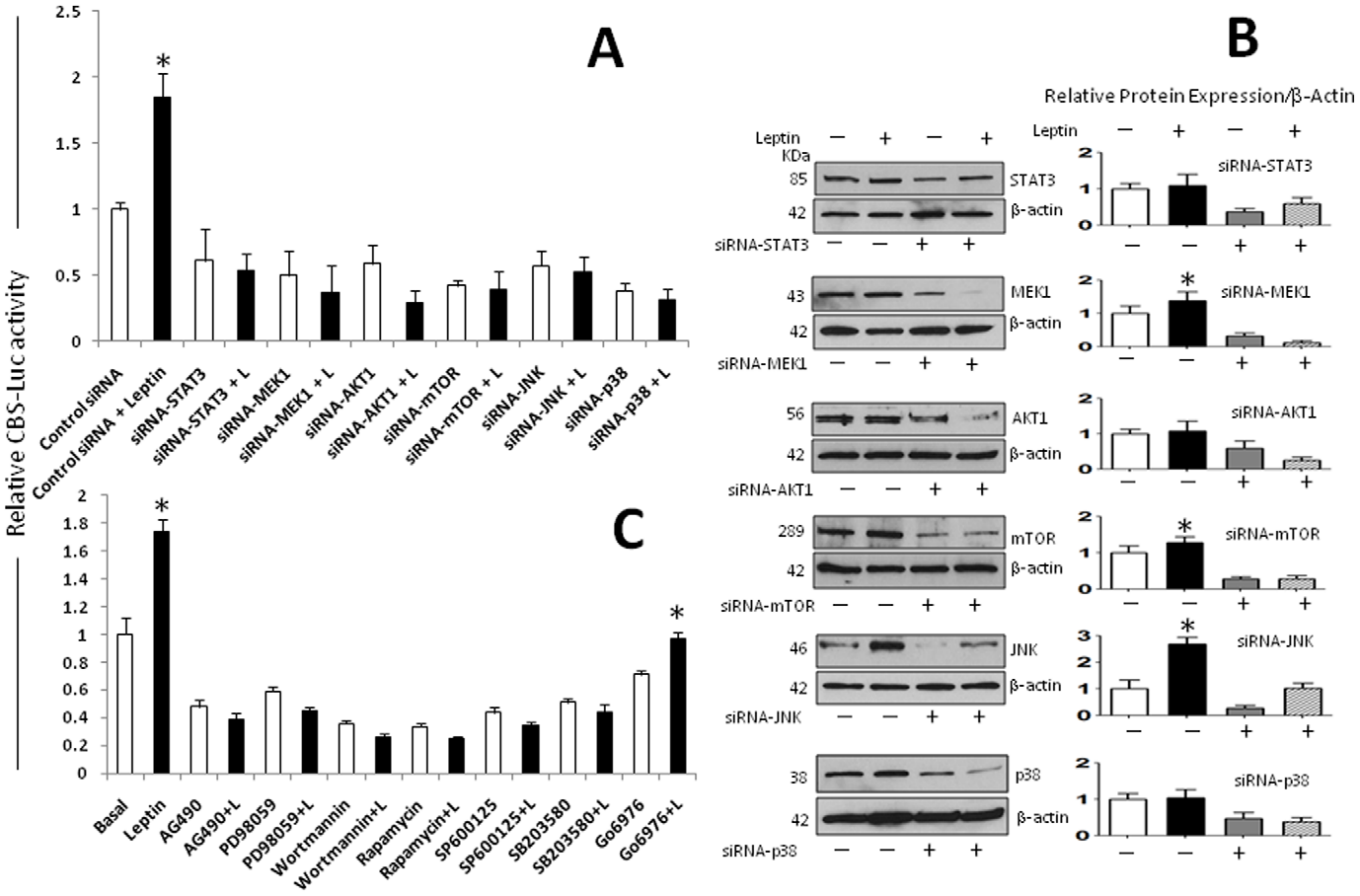
Leptin upregulates the CSL gene in mammary cancer cells. Leptin transcriptional activation of CBF (or CSL) promoter is linked to several leptin-induced signaling pathways. (A) 4T1 cells were transiently transfected with a CBF-Luc reporter construct and incubated with leptin (0 and 1.2 nM) alone or plus siRNA oligonucleotides (SignalSilence control-siRNA and STAT3, MEK1, AKT1, mTOR, JNK, p38-siRNA). (B) Protein levels of kinases after siRNA treatment were determined by WB analysis using β-actin as loading control. (C) 4T1 cells transfected with CBF-Luc reporter were incubated with leptin alone or plus pharmacological inhibitors of JAK2/STAT3 (AG490, 30 µM), MEK/MAPK/ERK1/2 (PD98059, 30 µΜ), PI-3K/AKT1 (Wortmannin, 50 nM), PKC-Ca dependant (Gö6976, 30 µΜ), p38 kinase (SB203580, 2 µΜ), JNK (SP600125, 30 µM) and mTOR (Rapamycin, 20 nM) signaling pathways for 24 h. Luciferase activity was determined as described (see M &M) and expressed as a percent of basal or leptin-treated cells. (a) *P<0.05* when comparing levels of luciferase activity to control (basal) or leptin-treated cells. Data (mean ± standard error) representative results derived from a minimum of 3 independent experiments.

To investigate if leptin upregulates CSL (CBF) transcription, 4T1 cells were co-transfected with CSL-reporter-luciferase and *Renilla* control-plasmid (pGL3-CBF). As expected, leptin significantly induced firefly luciferase expression in CBF-Luc cells (Figure 4). Next, to investigate which leptin-induced signals regulate CSL, cells were treated with leptin and leptin plus siRNA and pharmacological inhibitors of main leptin-induced kinases. Treatment of cells with leptin and siRNA for STAT3, MEK1, AKT1, mTOR, JNK and p38 MAPK significantly reduced basal levels and leptin-mediated increase of CSL-Luc levels (Figure 4A). WB analysis was used to assess whether siRNA treatment effectively reduced the expression of kinases after 24 h (Figure 4B). To further assess the role of leptin-induced kinases on CSL expression several pharmacological inhibitors were used. Inhibitors of canonic JAK2/STAT; MAPK/ERK 1/2 and PI-3K/AKT and non-canonic signaling pathways JNK and p38 MAP kinases completely abrogated leptin-induced up-regulation of CBF-Luc (Figure 4C).

### Effects of Notch inhibition on leptin-induced breast cancer cell proliferation and migration

**Figure 5:**
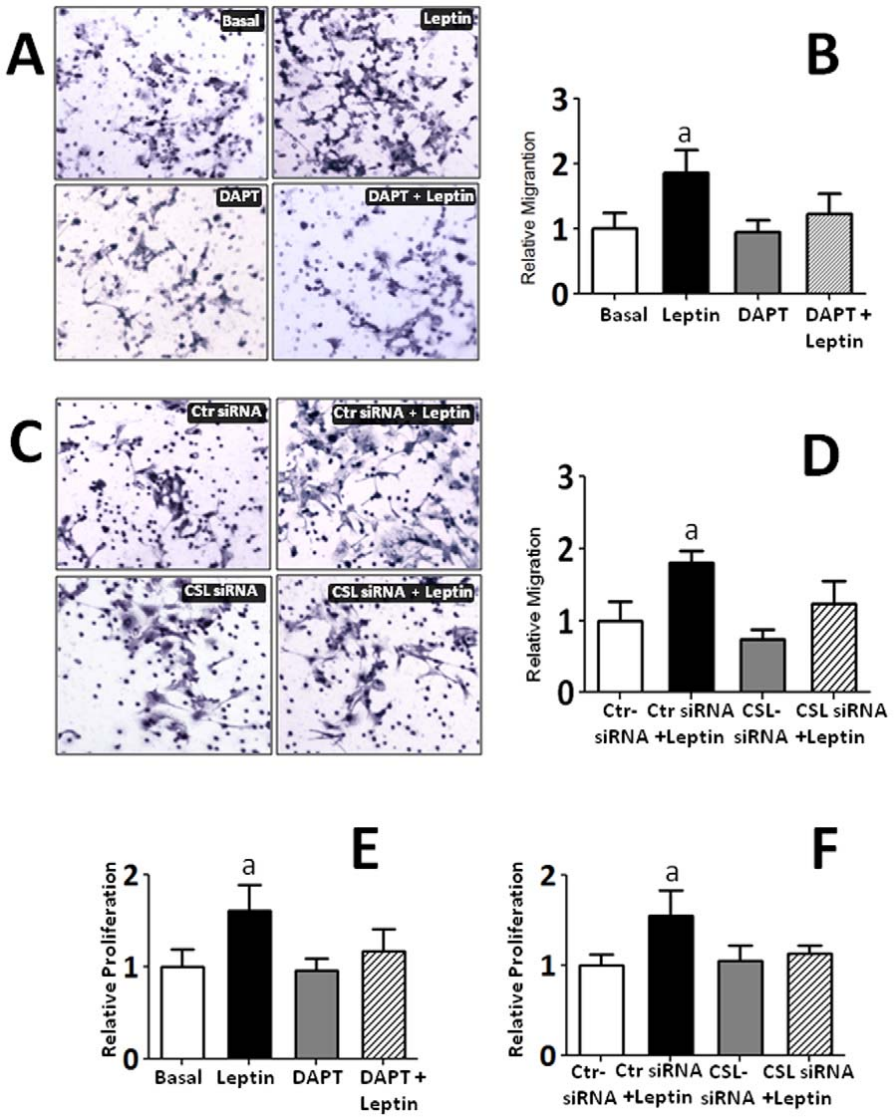
Leptin-induced 4T1 cell migration and proliferation was abrogated by Notch inhibition. A) Representative results from immunocytochemistry (ICC, hematoxylin staining) of leptin and DAPT effects on migration of 4T1 cells as compared to basal conditions. B) Quantitative assessment of cell migration under the effects of leptin and DAPT. C) Representative results from ICC after addition of control-siRNA (Ctr-siRNA), CLS-siRNA and leptin. D) Quantitative assessment of cell migration under the effects of leptin, CSL-siRNA and Crt-siRNA. E) Effects of leptin and DAPT on 4T1 cell proliferation. F) Effects of leptin, Ctr-siRNA and CSL-siRNA on 4T1 cell proliferation. Results from cell migration (Boyden chamber cell migration assay) and proliferation (MTT cell proliferation assay) were obtained after 24 h and normalized to basal conditions (see Material and Methods). Data (mean ± standard error) representative results derived from a minimum of 3 independent experiments.

To explore the biological significance of leptin-Notch crosstalk in breast cancer, we addressed the question whether the reported induction of cell proliferation and migration by leptin is affected by inhibition of Notch activation (DAPT; a γ-secretase inhibitor) and/or down regulation of its DNA-binding partner (CSL gene knockdown using CSL-siRNA). Figure 5 shows leptin significantly induced migration (Fig 5A-D) and proliferation of 4T1 cells in vitro (Fig 5E–F). Remarkably, these leptin effects were completely abrogated by inhibition of Notch signaling either with DAPT or CSL-siRNA. Furthermore, the leptin-induction of Notch proteins/activation paralleled the leptin-mediated induction of proliferation/migration of breast cancer cells. In contrast, DAPT alone and control-siRNA has no significant effects on basal or leptin-induced 4T1 cell migration or proliferation (see Figure 5A–F).

### Leptin-induced Notch regulates the levels of pro-angiogenic and pro-inflammatory factors

**Figure 6:**
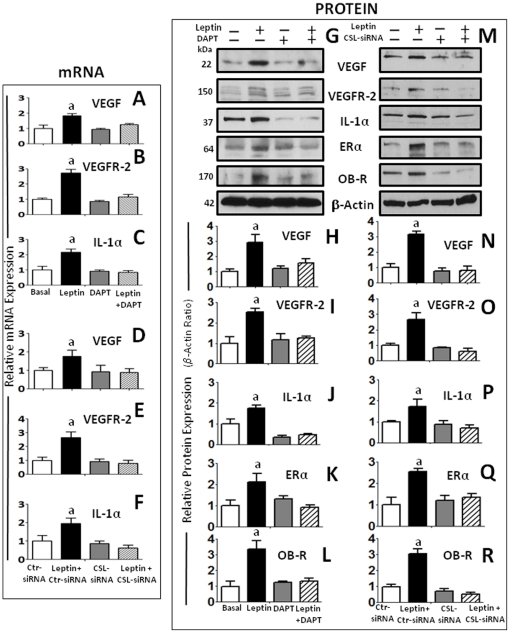
Leptin-Notch crosstalk regulates leptin-induced levels of pro-angiogenic and pro-inflammatory factors in mammary cancer cells. (A) DAPT inhibition of γ-secretase abrogated leptin transcriptional induction of VEGF (A); VEGFR-2 (B) and IL-1α mRNA (C) as determined by real-time RT-PCR and normalized to the GAPDH expression. CSL-siRNA also inhibited leptin-induced effects on protein levels of VEGF (D); VEGFR-2 (E) and IL-1α (F) mRNA. Representative results of DAPT (G) and CSL-siRNA (M) inhibition of leptin upregulation of protein levels of VEGF, VEGFR-2, IL-1α, ERα and OB-R in 4T1 cells as determined by western blot (WB). The WB results were normalized to β-actin as loading control and densitometric analysis of bands was carried-out with the imageJ software for VEGF (H and N); VEGFR-2 (I and O); IL-1α (J and P); ERα (K and Q) and OB-R (L and R) in cells treated with leptin (0 and 1.2 nM) and DAPT for 24 h or cotransfected with CSL-siRNA and control (Ctr)-SiRNA, respectively. (a) *P<0.05* when comparing levels of antigens to basal conditions and control-siRNA. Data (mean ± standard error) representative results derived from a minimum of 3 independent experiments.

Leptin regulates the transcriptional (Figure 6A–F) and translational (Figure 6G–J and Figure 6M–P) expression of VEGF/VEGFR-2 and IL-1α in 4T1 cells as determined by RT-PCR and western blot (WB), respectively. In addition, leptin upregulates the protein levels of ERα and OB-R. (Figure 6M, Q and R). Moreover, we further determined that inhibition of Notch activation (by DAPT) and CSL gene knockdown (by CSL-siRNA) abrogated the leptin-induced effects on the above described molecules at mRNA (Figure 6A–F) and protein levels (Figure 6G–R). The effects of leptin, DAPT and CSL-siRNA on IL-1α and VEGF levels in cell culture supernatants were further assessed by ELISA. IL-1α and VEGF levels showed similar patterns (data not shown) than those found by WB analysis.

### Inhibition of IL-1R tI signaling negatively impacted on leptin upregulation of Notch and target genes in breast cancer cells

**Figure 7:**
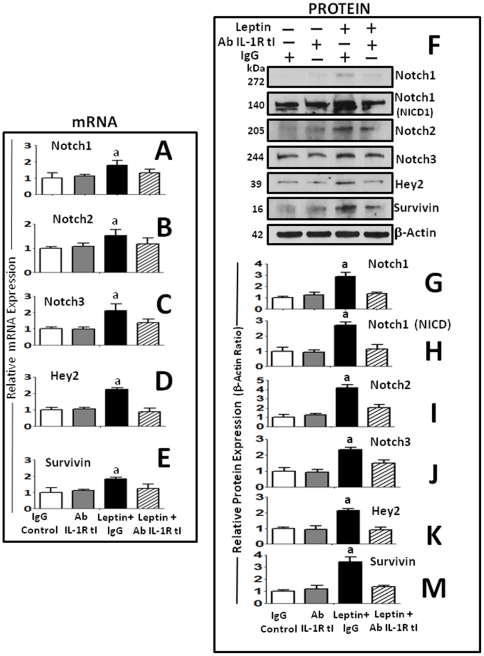
Inhibition of IL-1R tI signaling negatively impacts on leptin-mediated upregulation of Notch receptors and target genes in mammary cancer cells. Anti-IL-1R tI antibodies abrogated leptin transcriptional induction of Notch1 (A); Notch2 (B); Notch3 (C) and targeted genes, Hey2 (D) and survivin (E) in 4T1 cells as determined by real-time RT-PCR and normalized to the GAPDH expression. Blockade of IL-1R tI with antibodies also inhibited leptin-induced effects on protein levels of Notch and targeted genes. Representative results of IL-1R tI blockade on leptin upregulation of protein levels of Notch1, NICD1, Notch2, Notch3, Hey2) and survivin (F) as determined by western blot (WB). The WB results were normalized to β\-actin as loading control and densitometric analysis of bands was carried-out with the imageJ software for Notch1 (G); NICD1 (H); Notch2 (I); Notch3 (J), Hey2 (K) and survivin (M). 4T1 cells treated were treated with leptin (0 and 1.2 nM) and IL-1R tI antibodies or nonspecific species-matched IgG (Control Ab) for 24 h. (a) *P<0.05* when comparing Basal to Leptin+Control Ab and, (b) comparing Leptin+IL-1R tI Ab to Leptin+Control Ab. Data (mean ± standard error) representative results derived from a minimum of 3 independent experiments.

We further asked whether the blockade of IL-1R tI function also impacts Notch signaling pathway. Figure 7 shows that in contrast to control IgG, the blockade of IL-1/IL-1R tI signaling by IL-1R tI antibody significantly reduced leptin-induced transcriptional (Figure 7A–E) and translational (Figure 7F and Figure 7I–M) upregulation of Notch1, 2, 3, and Notch target genes, Hey2 and survivin in 4T1 cells. In addition, Notch1 NICD activation was also dependant of IL-1 signaling (Figure 7H).

## Discussion

Leptin is a growth and pro-angiogenic factor whose signals are strongly linked to the growth of solid tumors, particularly, breast cancer. We have previously shown that leptin induces growth and angiogenesis in mouse syngeneic mammary tumors [25], human breast cancer xenografts [26] and, in a mouse model for endometriosis-like lesions [28]. Leptin pro-angiogenic effects seem to be common in cancer since leptin differentially induces VEGF/VEGFR-2 in endometrial cancer, but not in normal cells [29]. Moreover, leptin induces VEGF/VEGFR-2 expression, angiogenesis and growth of human ovary cancer xenografts in nude mice and in 7,12- dimethylbenz[a]anthracene (DMBA)-diet-induced-obesity (DIO) mouse models of breast cancer (Gonzalez-Perez, Manuscript in preparation). Leptin-induced effects on VEGF/VEGFR-2 expression and growth of tumors/endometriotic tissues were abrogated by leptin signaling inhibition using antagonists of leptin receptor (PEG-LPrA) [25], [26], [28], [29]. Although all the mechanism(s) by which leptin contributes to tumor development are unknown, it appears leptin stimulates an increase in cell numbers, and the expression of VEGF/VEGFR-2.

We have recently described a comprehensive mechanism for leptin upregulation of VEGF in breast cancer [27]. Leptin uses several signaling pathways, including the activation of HIF-1α and NF-κB, for the upregulation of VEGF in breast cancer cells under normoxic conditions [27]. Moreover, leptin is a potent inducer of IL-1 system in breast cancer cells [18]. Interestingly, leptin pro-angiogenic signature in breast cancer was found linked to IL-1 signaling [18].

Notch, IL-1 and leptin are known pro-angiogenic inducers in breast cancer [3]. In addition, leptin and Notch are mitogenic factors for many cell types. Therefore, we hypothesized that their actions could be intimately related. Results from the present investigation strongly suggest that in addition of IL-1, leptin also upregulates Notch receptors, ligands and target genes. Furthermore, present results show that leptin-induction of cell proliferation and migration was abrogated by the inhibition of Notch. Moreover, leptin upregulation of VEGF/VEGFR-2 expression was highly dependent of a novel unveiled crosstalk between Notch, IL-1 and leptin (NILCO) in breast cancer cells.

Several genetic and pharmacologic approaches were used to determine how leptin regulates Notch (ligands/receptors/targets) and the impact of Notch signaling on leptin-induced VEGF/VEGFR-2 expression in breast cancer cells. Remarkably, leptin upregulates Notch at transcriptional/translational levels. Leptin signaling upregulates the activity of CSL-promoter and Notch mRNA expression as well as Notch activation. Leptin-induced canonical/non-canonical signaling pathways were involved in CSL promoter up-regulation. CSL (CBF or RBP-Jk) promoter activity was linked to leptin-activated JAK2/STAT3, MAPK, PI-3K/mTOR, p38 and JNK signaling pathways. Interestingly, leptin upregulation of Notch was abrogated by blockade of IL-1R tI. IL-1/IL-1R tI signals were previously found to mediate leptin upregulation of VEGF/VEGFR-2 in breast cancer [18].

**Figure 8:**
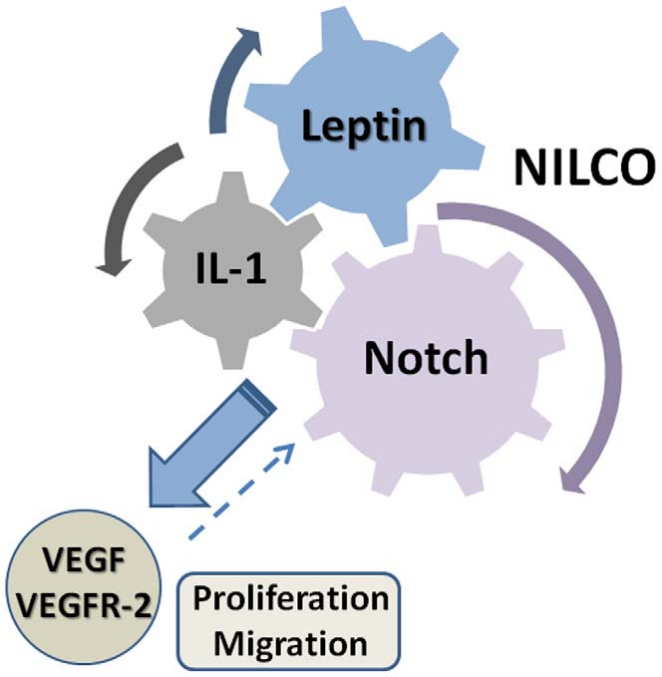
Notch, IL-1 and leptin crosstalk outcome (NILCO) upregulates VEGF/VEGFR-2 and mediates leptin-induced breast cancer cell proliferation/migration. Leptin up-regulates Notch and IL-1 in breast cancer cells [18]. Leptin can directly [27], or through IL-1 [18] and Notch, induce VEGF/VEGFR-2 up-regulation. VEGF signaling could also upregulate Notch [45]. Leptin-induced proliferation and migration of breast cancer cells was related to an intact Notch signaling axis. NILCO could be a master process for the regulation of breast cancer angiogenesis.

We have proposed that IL-1 could activate Notch signaling pathway through NF-κB activity [3]. In line with this hypothesis, Rel (a NF-κB subunit) was earlier identified as an activator of Notch1 signaling pathway by inducing expression of JAG1 [30], [31]. In addition, we have previously shown that leptin upregulates both IL-1 [18] and VEGF by NF-κB dependent mechanisms [27]. Present data suggest leptin upregulation of VEGF/VEGFR-2 was also mediated by leptin-induced Notch expression. These findings further suggest that leptin-induction of Notch and IL-1 plays an important role in leptin pro-angiogenic effects in breast cancer. Moreover, present findings show for the first time that leptin and Notch collaborate for the induction of cell proliferation/migration and that a complex signaling network between Notch, IL-1 and leptin (NILCO) is required for the upregulation of VEGF/VEGFR-2 in breast cancer (Figure 8).

Leptin has previously been identified as a pro-proliferation and anti-apoptotic factor that upregulates the expression of key molecules for cell cycle, Cdk2 and cyclin D1 [25], [26], [32], activity of Rb [33] and, apoptosis, Bcl-2 [34] and Bcl-x_L_ [35]. Present results showing leptin-mediated upregulation of Notch targets: survivin (a recently identified pro-survival factor) [36], [37] and Hey2 (a repressor and mediator of Notch) [38], and induction of cell proliferation and migration, further support the proliferative, pro-survival and anti-apoptotic effects of leptin.

On the other hand, leptin-mediated upregulation of Notch was found together with increased levels of IL-1α, ERα and OB-R. Interestingly, all these leptin-mediated effects were abrogated by inhibition of Notch activation or blockade of CSL expression. Leptin has previously shown to transactivate ERα [39] and to induce OB-R in breast cancer [18]. Moreover, Notch and ERα crosstalk in breast cancer [40]. However, to the best of our knowledge no previous reports on Notch-mediated upregulation of ERα are available. E2 promoted 8-fold and 6-fold increase in Notch1 and JAG1 expression, respectively, in breast cancer MCF-7 cells [40]. On the other hand, in ERα expressing cells, E2 inhibited Notch activity and affected NICD nuclear levels as well as Notch-1 cellular distribution [41]. Therefore, leptin-mediated induction of ERα through Notch signaling might represent a negative feed-back loop for Notch expression in breast cancer.

In conclusions, Notch-IL-1-Leptin crosstalk outcome (NILCO) is essential for leptin-induced proliferation/migration as well as the upregulation of VEGF/VEGFR-2 expression in breast cancer cells. Leptin-induced proliferation and migration effects as well as increased levels of pro-angiogenic factors in breast cancer cells were related to an intact Notch signaling axis. Overall, NILCO represents a novel crosstalk where signals from pro-angiogenic, pro-inflammatory and developmental factors converge to promote cell growth and tumor angiogenesis. NILCO could be a master regulator of pro-angiogenic factors in breast cancer and likely contribute to increase tumor angiogenesis and metastasic potential. Therefore, targeting this complex crosstalk could open the development of new combinatory therapeutic possibilities, in particular for the development of breast cancer linked to obesity and hyperleptinemia (*i.e.*, postmenopausal women). Combinatory treatments of PEG-LPrAs [42] with drugs designed to prevent Notch and IL-1 oncogenic signal crosstalk may be advantageous over drugs targeting individual players of NILCO.

## Materials and Methods

### Reagents and antibodies

Recombinant mouse leptin and mouse IL-1α/IL-1F1 and VEGF Quantikine ELISA Kits were purchased from R & D Systems (Minneapolis, MN). DAPT, N-[N-(3,5-difluorophenacetyl)-L-alanyl]-S-phenylglycine t-butyl ester, Monoclonal anti-Notch1, β-actin antibodies were purchased from Sigma-Aldrich. Fetal bovine serum (FBS) was obtained from Gemini Bioproducts (West Sacramento, CA), RPIM-1640 medium and penicillin-streptomycin cocktails were purchased from American Type Culture Collection (ATCC) (Manassas, VA). Polyclonal anti-Notch4, anti-OB-R-NH2 antibodies, monoclonal anti-IL-1R tI, nonspecific goat IgG2b, CSL small interference (si)RNA and control siRNA-A were obtained from Santa Cruz Biotechnology, Inc. (Santa Cruz, CA). STAT3, MEK1, AKT1, mTOR, SAPK/JNK and p38 MAPK-siRNA and, SignalSilence control siRNA were purchased from Cell Signaling (Danvers, MA). Polyclonal anti-Notch2, 3, anti-IL-1α antibodies were obtained from Abcam Inc (Cambridge, MA). Polyclonal anti-Hey2 was purchased from Millipore (Temecula, CA). Polyclonal anti-survivin and STAT3, MEK1, AKT1, p38 MAPK, SAPK/JNK antibodies were from Cell Signaling (Danvers, MA). Polyclonal anti-mTOR antibody was from Abcam (Cambridge, MA). Polyclonal anti-mouse antibodies, anti-rabbit horseradish peroxidase (HRP) conjugates, iScript cDNA Synthesis, IQ SYBR Green Supermix and protein determination kits were purchased from Bio-Rad Lab. (Hercules, CA). ECL Western blot stripping buffer was purchased from Thermo Scientific (Rockford, IL). Dual-luciferase assay system and pGL-3 plasmid were obtained from Promega (Madison, WI). Mouse IL-1α and VEGF ELISA Kits were purchased from R & D Systems (Minneapolis, MN)**.** RNeasy Mini kits, DNase kits and Superfect transfect reagents were obtained from Qiagen (Valencia, CA). AG490, PD98059, Wortmannin, Gö6976, SB203580, SP600125, Rapamycin, Endofree plasmid maxiprep kit, protease inhibitor and phosphatase inhibitor cocktails 1 and 2 and other chemicals were purchased from Sigma-Aldrich (St. Louis, MO).

### Cell culture

The mouse mammary tumor (MT) cell line 4T1 (CRL-2539; ATCC), EMT6 (CRL-2755) and MMT060562 (MMT; CCL-51) were cultured with complete RPMI-1640 containing 10% fetal bovine serum (FBS), 100 units/ml penicillin and 100 µg/ml streptomycin on uncoated flat-bottomed plastic plates (cell densities of 1.0, 2.0 or 4.0×10^5^ cells/well for 24, 12 or 6 well plates). Semi-confluent cells were starved for 24 h in basal medium (RPMI-1640 without FBS) and treated with different compounds. In all experiments triplicate wells, tubes and reactions were run and repeated at least three times with different cell preparations.

### Leptin dose-response effects

MT cells were starved as described above and incubated for 24 h with medium containing leptin (0, 0.6, 1.2, and 6.25 nM, equivalent to 0, 10, 20 and 100 ng/ml). Proteins and mRNAs were determined by western blot and real-time RT-PCR, respectively. Protein concentrations in cell lysates were determined by the Bradford method (Bio-Rad Lab).

### Immunocytochemistry (ICC)

To assess leptin effects on the expression of Notch molecules and their targets 4T1 cells (5×10^5^ cells/chamber) were cultured in ICC-treated glass slides (BD FalconTM, Belford, MA) until 80% confluence and incubated with 1.2 nM leptin for additional 24 h. The slides were fixed in 10% formalin (3.7% formaldehyde in PBS) for 24 h and incubated with antibodies for Notch1∼4, DLL-4, Hey2 and survivin for 4 h. Negative controls were made by omitting primary antibodies. Mouse and rabbit ABC staining systems (Santa Cruz, CA) were used according to the manufacturer's instructions. Slides were counterstained with Mayer's hematoxylin and mounted with cover slips using an aqueous mounting media. Images were obtained in a Leica TCS Confocal microscopy at x200 magnifications.

### Specific kinases involved in leptin-mediated effects

4T1 cells were starved as described above and incubated with 1.2 nM leptin and kinase inhibitors (AG490 for JAK2/STAT3, 30 µM; PD98059 for MEK/MAPK/ERK1/2, 30 µΜ; Wortmannin for PI-3K/AKT1, 50 nM; Gö6976 for PKC-Ca dependant, 30 µΜ; SB203580 for p38 kinase, 2 µΜ; SP600125 for JNK, 30 µM; and Rapamycin 20 nM for mTOR) for 24 h.

### Reporter gene transfection and luciferase assay

Semi-confluent 4T1 cells were transiently cotransfected using superFect transfection reagent (Qiagen) with 50 ng of a *Renilla* reporter-luciferase control-plasmid and 500 ng of pGL3-CBF plasmid containing firefly luciferase reporter gene (kindly provided by Dr.Yizeng Yang, University of Pennsylvania). After 3 h cotransfection, the cells were incubated with 1.2 nM leptin for 24 h. Luciferase activity in cell lysates was determined using the dual-report assay system (Promega) according to the manufacturer's instructions. Normalization was based on cotransfected *Renilla* luciferase activities.

### RNA extraction and real-time PCR

RNA was extracted from 4T1 cells using RNeasy Mini Kit (Qiagen). First-strand cDNA was synthesized from total RNA using SuperScript First-Strand Synthesis System with SuperScript II reverse transcriptase according to the manufacturer's protocols (Invitrogen, Carlsbad, CA). The cDNA was used as a template in real-time PCR reactions with QuantiTect SYBR-Green PCR master-mix (Bio-RAD) and was run on an ABI PRISM 7700 machine. Real-time quantitative PCR reactions consisted of 1x SybrGreen Supermix (Bio-Rad), 0.25mmol/L forward and reverse primers, and 10 ng cDNA. Cycling conditions consisted of a three-step amplification and melt curve analysis using the iQ5 Real-time PCR Detection System (Bio-Rad). For generating a standard curve, amplified cDNA from the reference sample detailed above was used in a 5-fold dilution series of 100 to 0.16 ng cDNA per reaction. Relative gene expression was calculated by dividing the specific expression value (starting quantity, ng) by the glyceraldehyde-3-phosphate dehydrogenase (GAPDH) expression value. Primers used in the experiment were: Mouse Notch1 forward: 5′-tgttgtgctcctgaagaa cg-3′ and reverse: 5′-tccatgtgatccgtgatgtc-3′; Mouse Notch2 forward: 5′-gaggcgactcttctgctgtt-3′ and reverse: 5′-ggtccatgtggtcagtgatg-3′; Mouse Notch3 forward: 5′-caatgcagtggatgagcttg-3′ and reverse: 5′-ccaagaacagcggcgtct-3′; Mouse Notch4 forward: 5′-ccgctgctgtgaacaacg-3′ and reverse: 5′-cacctccacggctccttc-3′; Mouse Jagged1 forward: 5′-atgatgggaaccctgtcaag-3′ and reverse: 5′-cagagctcagcagaggaacc-3′; Mouse CSL forward: 5′-caatgcttgaacttacaggaca-3′ and reverse: 5′-ggacccatctccaaccttc-3′; Mouse survivin forward: 5′-gcggaggctggcttca-3′ and reverse:

5′-agaaaaaacactgggccaaatc-3′; Mouse Hey2 forward: 5′-cctgtctcccaggctacact-3′ and reverse: 5′-ggcagtggtagctattctcctg-3′; Mouse DLL-4 forward: 5′-agctgggtgtctgagtaggc-3′ and reverse: 5′-agaaggtgccacttcggtta-3′; Mouse IL-1α forward: 5′-tcgggaggagacgactctaa-3′ and reverse: 5′-aggtcggtctcactacctgtg-3′; Mouse VEGFR-2 forward: 5′-gtgattgccatgttcttctggc-3′ and reverse: 5′-ttcatctggatccatgacaa-3′; Mouse VEGF forward:5′-tacctccaccatgccaagtggt-3′ and reverse:5′-aggacggcttgaagatgtac-3′. The GAPDH was used as internal control using the following primers: forward: 5′-tgcaccaccaatgcttag-3′ and reverse: 5′-ggatgcagggatgatgttc-3′.

### Western blot (WB)

Following cytokine and antibody treatment, cells were washed once with ice-cold PBS and disrupted by homogenization in RIPA buffer (Sigma) contained 0.1 mg/ml protease inhibitor, 1∶100 phosphatase inhibitor cocktail 1 and 2. Cellular lysates were centrifuged for 10 min at 24,000 g/4°C. Twenty-µg proteins were loaded per lane and electrophoresis/electroblotting using specific antibodies. Protein-bands were detected using SuperSignal West Pico Chemiluminescent Substrate (Pierce) and exposed on Kodak X-OMAT film (Kodak). Quantitative WB data were calculated from densitometric analysis of bands with the NIH imageJ software. The values were normalized to β-actin as loading control.

### Determination of IL-1 and VEGF levels by ELISA

Semi-confluent untransfected 4T1 (treated with DAPT; 5 uM/0.1% DMSO final concentration) or transfected with siRNA oligonucleotides were incubated with 1.2 nM leptin for 24 h (as described above). Culture supernatants were used to assess leptin, DAPT and CSL-siRNA effects on the levels of IL-1α and VEGF proteins as determined by ELISA.

### CSL-RNA knockdown

2.0×10^5^ 4T1 cells were cultured in 6-well plates were starved as described above and cotransfected with siRNA oligonucleotides (CSL-siRNA and control) using Superfect reagent (Qiagen). After 4 h of transfection, the medium was changed to complete medium containing 1.2 nM leptin for 24 h. Then, levels of Notch receptors and targeted proteins were determined by WB analysis as described above.

### Leptin-induced kinase RNA knockdown and CSL-luciferase assay

0.5×10^5^ 4T1 cells were cultured in 24-well plates and starved as described above. Then, cells were cotransfected with 50 ng of a *Renilla* reporter-luciferase control-plasmid and 500 ng of pGL3-CBF (CSL) plasmid as well as siRNA oligonucleotides (SignalSilence control-siRNA and STAT3, MEK1, AKT1, mTOR, JNK, p38-siRNA) using Superfect reagent (Qiagen). After 4 h of transfection, the medium was changed to complete medium containing 1.2 nM leptin for 24 h. Then, luciferase activity was determined as described above. Protein levels of kinases after siRNA treatment were determined by WB analysis using β-actin as loading control.

### MTT cell proliferation assay

MT cells were seeded 1×10^4^ per well in a 96-well plate. Cells were starved as described above and incubated for 24 h in medium containing 0 and 1.2 nM leptin plus 0.1% dimethylsulfoxide (DMSO), DAPT, control-siRNA and CSL-siRNA. Then, 20 µl of 3-(4,5-dimethylthiazol-2-yl)-2,5-diphenyltetrazolium bromide (MTT) (5 mg/ml) was added to each well. After 4 hours of incubation at 37°C, cells were lysed by addition of 200 µl DMSO. Absorbance was measured at 570 nm using a microplate reader (Molecular Devices, CA).

### Boyden chamber cell migration assay

Migration assay was performed as previously described [43], [44]. 4T1 cells (5×10^4^) suspended in starvation medium were added to the upper chamber of an insert (6.4-mm diameter, 8-µm pore size; BD Biosciences). *- Inhibition of γ-secretase:* cells in the upper chamber were suspended in basal medium plus 0.1% DMSO (Basal) or DAPT (5 uM DAPT/0.1% DMSO) and the insert was placed in a 24-well plate containing starvation medium with or without 1.2 nM leptin. *-CSL knockdown:* Cells were previously transfected for 4 h with Ctr-siRNA or CSL-siRNA and then placed in the upper chamber. Then, the insert was placed in a 24-well plate containing 0 or 1.2 nM leptin. Migration assays were carried out for 24 h. Then, the cells in the lower side of the insert were fixed with 3.7% formaldehyde. Cells were stained with hematoxylin and cells on the upper side of the insert were removed with a cotton swab. Six randomly selected fields (X10 objective) were photographed, and the migrated cells were counted.

### Blockade of IL-1R tI

4T1 cells were starved as described and incubated with basal medium containing 1.2 nM leptin and 0.1 µg/ml anti mouse IL-1R tI antibody for 24 h which we described previously [18]. Nonspecific species-matched IgG2b served as negative controls. Several proteins in cell lysates were analyzed by WB.

### Data analysis

*Student'*s *t*-test was used for data analysis. Data (mean ± standard error) representative results derived from a minimum of 3 independent experiments. Values for *p*<0.05 were considered statistically significant. The model included the main effects of treatments and replicates.
